# QTL controlling grain filling under terminal drought stress in a set of wild barley introgression lines

**DOI:** 10.1371/journal.pone.0185983

**Published:** 2017-10-20

**Authors:** Nora Honsdorf, Timothy J. March, Klaus Pillen

**Affiliations:** Plant Breeding, Institute of Agricultural and Nutritional Sciences, University of Halle-Wittenberg, Halle/Saale, Germany; Institute of Genetics and Developmental Biology Chinese Academy of Sciences, CHINA

## Abstract

Drought is a major abiotic stress impeding the yield of cereal crops globally. Particularly in Mediterranean environments, water becomes a limiting factor during the reproductive developmental stage, causing yield losses. The wild progenitor of cultivated barley *Hordeum vulgare* ssp *spontaneum* (*Hsp*) is a potentially useful source of drought tolerance alleles. Wild barley introgression lines like the S42IL library may facilitate the introduction of favorable exotic alleles into breeding material. The complete set of 83 S42ILs was genotyped with the barley 9k iSelect platform in order to complete genetic information obtained in previous studies. The new map comprises 2,487 SNPs, spanning 989.8 cM and covering 94.5% of the *Hsp* genome. Extent and positions of introgressions were confirmed and new information for ten additional S42ILs was collected. A subset of 49 S42ILs was evaluated for drought response in four greenhouse experiments. Plants were grown under well-watered conditions until ten days post anthesis. Subsequently drought treatment was applied by reducing the available water. Several morphological and harvest parameters were evaluated. Under drought treatment, trait performance was reduced. However, there was no interaction effect between genotype and treatment, indicating that genotypes, which performed best under control treatment, also performed best under drought treatment. In total, 40 QTL for seven traits were detected in this study. For instance, favorable *Hsp* effects were found for thousand grain weight (TGW) and number of grains per ear under drought stress. In particular, line S42IL-121 is a promising candidate for breeding improved malting cultivars, displaying a TGW, which was increased by 17% under terminal drought stress due to the presence of an unknown wild barley QTL allele on chromosome 4H. The introgression line showed a similar advantage in previous field experiments and in greenhouse experiments under early drought stress. We, thus, recommend using S42IL-121 in barley breeding programs to enhance terminal drought tolerance.

## Introduction

Drought is a major abiotic stress impeding the yield of cereal crops globally. Limitations in soil water availability during both the vegetative and reproductive stages of plant development can result in yield losses. However, it is often the case, particularly in Mediterranean environments, that water becomes limiting during the reproductive developmental stage, causing yield losses through reducing both grain number and grain size.

Drought stress at the beginning of the reproductive stage can effect floral meristem differentiation and reduce the number of spikelets, and, thus, eventually the number of grains per spike. Once formed, the florets within each spikelet are also particularly sensitive to drought stress as they go through gametogenesis, leading to sterility and loss of seed set [1]. Following anthesis, the main impact of drought stress is to reduce grain size and weight. This can primarily occur in one of two ways, either through limiting the number of endosperm cells formed and hence the potential size of the grain, or by reducing the rate and duration of starch accumulation in the endosperm.

After fertilisation, the caryopsis undergoes cell differentiation. During this time, the final cell number of the mature grain is determined. Drought stress during this early stage has been shown to reduce the mitotic division of the endosperm cells by up to 90%, consequently restricting the final grain size [2]. In barley, at approximately ten days post-anthesis (DPA) starch granules are first observed within the endosperm cells and they continue to grow until approximately 45 DPA [3,4]. The rate of starch granule growth is reduced under drought stress due to a limited supply of assimilates into the grain. Under optimal growth conditions assimilates are sourced from current photosynthesis, however, under drought stress premature senescence reduces this source and the main carbon source for grain filling comes from remobilising carbohydrates stored in the stem [5]. Therefore, the plants ability to accumulate and then remobilise stem carbohydrates under drought stress is a proposed mechanism to maintain grain filling under drought conditions. Also, physiological responses may enhance drought tolerance. In this regard, hormonal homeostasis, in particular between brassinosteroids, auxins, cytokinins, gibberellins and abscisic acid proved to be important [6]. In addition, the osmotic adjustment of drought tolerant genotypes may be achieved through accumulation of osmotic active compounds such as proline [7].

The key to improving the level of drought tolerance in cereals is through identifying improved sources of genetic variation. Several studies have now highlighted that the wild progenitor of cultivated barley *H*. *vulgare* ssp *spontaneum* (hereafter abbreviated *Hsp*) is a potentially useful source of drought tolerance alleles [8–10]. Since *Hsp* can be crossed with cultivated barley, once tolerance alleles are identified, they can be directly used by breeders [11].

One limitation in using wild species in genetic studies is their undesirable agronomic attributes that can mask the presence of favourable alleles, although this issue can be overcome using an advanced backcross QTL strategy [12]. In barley the advanced backcross QTL approach has been used successfully to identify a range of *Hsp* derived alleles for improved agronomic performance, malt quality, disease resistance [13–17] and vegetative drought tolerance [18,7].

A further tool to make use of exotic germplasm in plant breeding are introgression line (IL) libraries [19]. In contrast to AB populations, the individuals harbor only one (or very few) introgression. Schmalenbach et al. [20] developed the IL library S42IL from 40 genotypes of the advanced backcross QTL population S42 (BC_2_DH) developed by von Korff et al. [21]. The parents used for the development of the population are the malting barley cultivar Scarlett and the *Hsp* accession ISR42-8. Through multiple rounds of backcrossing and marker assisted selection, a set of 73 S42ILs was developed. Each individual S42IL contains on average 3.3% of the *Hsp* donor genome and collectively the library represents 87.3% of the *Hsp* donor genome [22]. Later the library was extended and now comprises 83 introgression lines. Subsets of S42ILs have been studied in a wide range of studies including experiments under field, greenhouse, and hydroponic conditions, different watering and fertilizing treatments, and different growth stages [20,22–30].

Recently, also multiparental population designs were developed in barley to cross the bridge between cultivated and wild barley gene pools. For this, two nested association mapping (NAM) populations [31–33] and one multi-parent advanced generation inter-cross (MAGIC) population were developed. These populations include *Hsp* derived wild barley and old barley land races, respectively, and were used to study exotic barley alleles, which potentially improve agronomic traits in cultivated barley.

In this current study we aim to identify genetic diversity for terminal drought tolerance in a subset of 49 wild barley introgression lines of the S42IL library [22]. The identification of useful phenotypic variation for terminal drought tolerance in the S42IL library will facilitate the rapid map-based cloning of the identified QTL. Moreover the complete set of 83 42ILs was genotyped with the barley 9k iSelect platform [34] in order to obtain a dense characterization of the complete S42IL set.

## Materials and methods

### Plant material

In this study, a subset of 49 S42ILs was evaluated for drought tolerance. Moreover, the complete set of 83 S42ILs was genotyped with the barley 9k iSelect platform [34] in order to construct a map of the entire library. The development of the S42IL population has been described in detail previously [20,22]. Briefly, the *H*. *vulgare* ssp. *vulgare* German malting variety 'Scarlett' and the *H*. *vulgare* ssp. *spontaneum* accession 'ISR42-8' were used to produce a BC_2_ doubled haploid population with Scarlett as the recurrent parent [21]. Then a further round of back-crossing to Scarlett and several rounds of self-pollination, combined with marker-assisted selection, were carried out to select a set of lines that individually contain a single introgression from ISR42-8 in an otherwise Scarlett background, but collectively represent the entire ISR42-8 genome.

### Genotyping

Genomic DNA was extracted from 30–50 mg leaf material, which was pooled from twelve seedling plants per genotype. To each sample, 400 ml of RLT buffer (Qiagen, Hilden, Germany) were added and samples were disrupted using a TissueLyser bead mill (Qiagen). DNA was extracted using Qiagen´s BioSprint DNA Plant Kit and the BioSprint 96 workstation. DNA was dissolved in distilled water. The samples were sent to Trait Genetics, Gatersleben, Germany (http://www.traitgenetics.com) and genotyped with the barley 9k iSelect platform [34]. Obtained raw data were transformed into genotype calls. SNP positions for map construction were taken from Comadran et al. [34]. SNPs which were not polymorphic between the parents Scarlett and ISR42-8, had no genotype or map position were removed from the set.

### Drought stress treatment

The set of 49 S42ILs and Scarlett as the control genotype were grown under glasshouse conditions. Ten plants per genotype were grown together in a 1.5 L free draining pot, filled with a cultivation substrate containing peat, clay, and NPK fertilizer with 250, 300, and 400 mg/L nitrogen, phosphate, and potassium, respectively, at pH 5.8. The high number of plants per pot was used to reduce tillering so that only primary tillers were studied. Plants were grown under a 14/10 hour day/night photoperiod with a 24/18°C day night average temperature. The experiment was arranged in a split-plot design. The genotypes were randomly assigned to whole-plots within each experimental block, where each whole-plot consisted of two pots of the same genotype. Watering treatments (well-watered or drought) were then assigned to one of the two pots (sub-plots) within each whole-plot. Each experiment comprised three complete blocks arranged in a single glasshouse room. In total four independent experiments were conducted, giving a total of twelve experimental blocks.

Plants were watered daily with an automated drip irrigation system. Prior to the drought treatment, each pot was watered to a field capacity of 70%. At ten days post-anthesis, the drought and well-watered pots were supplied with 30 mL and 90 mL of water, respectively, until maturity. Measurements of random pots showed this watering level resulted in a field capacity of approximately 10–20%, while the well-watered pots remained at a field capacity of 70%.

### Plant phenotyping

The plants were phenotyped for a range of morphological parameters. Days to heading (HEA) was recorded when 5 cm of awns protruded from 50% of the ears in each pot. Plant height (HEI) was measured as the average distance between the soil and the base of the ear at maturity. At the completion of the experiment, mature ears from the main stems were harvested and threshed using a homemade mechanical threshing machine. The following yield parameters were then evaluated; grain yield (YLD) was measured as the total grain weight harvested from an individual pot, the number of grains per ear (GEA) was determined by dividing the total grain number harvested per pot by the number of harvested ears. Grain weight per ear (GWE) was calculated by dividing YLD by the number of ears harvested per pot. Thousand grain weight (TGW) was determined by dividing the pot YLD by the number of harvested grains per pot and multiplying by 1000. Plant biomass (BIO) was measured by weighing total above ground vegetative biomass of each pot (excluding ears) after two days drying at 80°C.

### Statistical analyses

Statistical analysis of the split-plot drought experiment was performed in the R statistical environment [35]. For each trait a linear mixed-model was fitted using the package ASReml-R [36]. The model consisted of the following terms: y_(*ijk*)_ = mean + genotype_(*i*)_ + treatment_(*j*)_ + genotype*treatment_(*ij*)_ + block_(*k*)_ + block*genotype_(*ki*)_ + error. Terms in bold type were treated as random effects, while all others were fixed effects. For the traits HEI and HEA, the ‘treatment’ term was removed from the model since the HEI and HEA measurements were averaged across the treatment sub-plots before the treatment began. From the model predicted means were obtained for each combination of the genotype and treatment levels. Pair-wise confidence intervals were then calculated to identify S42ILs significantly different to Scarlett as the control genotype (p ≤ 0.05). If an individual S42IL was significantly different to Scarlett in only one treatment or both treatments it was classed as a line by treatment or line main effect respectively. When an S42IL showed a significant difference to Scarlett, it was assumed that a QTL was present within the *Hsp* introgression of that line, if two independent S42ILs contained overlapping *Hsp* introgression, and both lines exhibited a significant trait effect with the same sign, it was assumed they contained the same QTL. Pearson’s correlation coefficient (r) was calculated between the predicted means of each trait in R using the function r*corr* in the R package *Hmisc*.

## Results

### Genotyping

**Fig 1:**
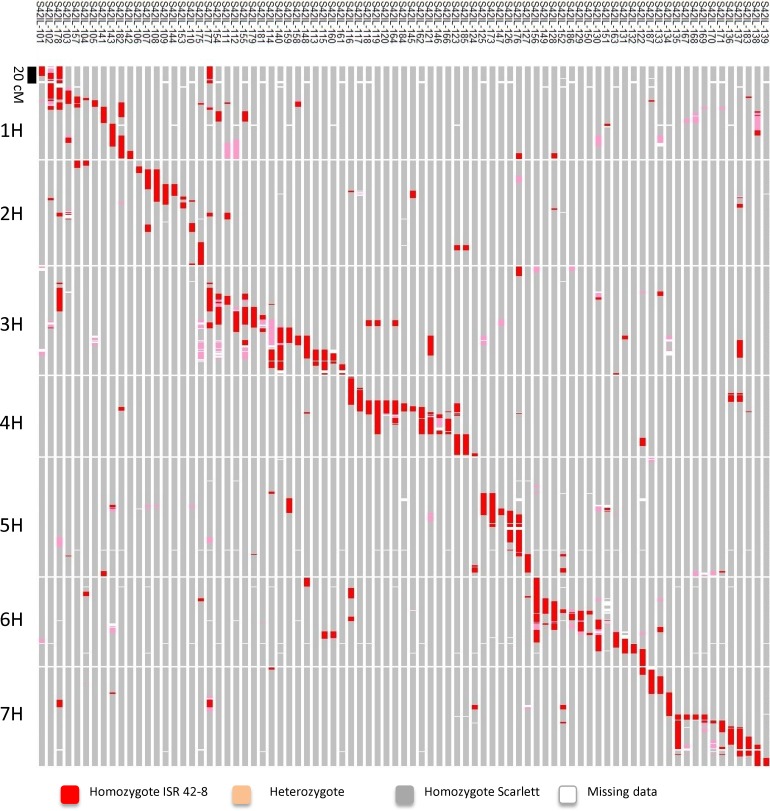
Map of 83 S42ILs containing 3,059 SNP markers.

Eighty-three S42ILs were genotyped with the Infinium 9k iSelect assay. The assay comprises 7,864 SNPs. For map construction only SNPs were included which were polymorphic between the parents Scarlett and ISR42-8. Moreover, marker data were manually curated and unlikely double crossovers were removed. This resulted in 2,487 SNP markers that were used to construct a new map of the complete S42IL library. The map is 989.8 cM long with 94.5% of *Hsp* genome represented by all 83 S42ILs together. On average, each S42IL contains 11.3 cM of the *Hsp* genome, but sizes of main introgressions vary from 2.5 to 91.4 cM (Fig 1, Table 1, S1 Table). Fifteen S42ILs harbor a single introgression according to the iSelect based map. The remaining lines possess between one and seven introgressions in addition to the target introgression. Among those, the majority possess one (29 lines) or two (19 lines) additional *Hsp* introgressions. The share of heterozygous SNP loci per genotype varies between 0 and 2.7%. Missing values per genotype range between 0.1 and 3.3%.

**Table 1:** Genetic characterization of position and extent of *Hsp* introgressions of 83 S42ILs based on the Infinium 9k iSelect assay.

Chr.	S42IL	Pos. of first SNP[cM]^1^	Pos. of last SNP [cM]	Min. size of target intr. [cM]	Hsp %^2^	Het %	Missing %	No. add. intr.
1H	S42IL-101	0.2	12.5	12.3	0.7	2.5	1.0	4
	S42IL-102	0.2	62.3	62.1	5.9	1.6	2.3	2
	S42IL-178	0.2	46.6	46.4	11.7	2.1	0.6	7
	S42IL-103	32.2	53.7	21.5	4.9	0.7	1.3	4
	S42IL-157	43.2	58.4	15.2	5.0	0.2	0.4	1
	S42IL-104	46.3	48.8	2.5	3.2	0.2	0.3	2
	S42IL-105	48.1	57.3	9.2	3.0	0.7	0.3	1
	S42IL-141	58.4	80.2	21.9	2.1	0.1	0.3	1
	S42IL-143	82.6	112.3	29.6	2.2	1.6	1.0	3
	S42IL-182	100.8	130.8	30.0	3.8	0.6	0.3	2
	S42IL-142	122.1	132.7	10.6	0.9	0.0	0.1	0
2H	S42IL-106	8.9	17.6	8.7	0.8	0.0	0.2	0
	S42IL-107	12.5	41.2	28.7	2.9	0.1	0.2	2
	S42IL-108	12.5	59.1	46.5	7.0	0.1	0.1	1
	S42IL-109	33.9	62.7	28.9	6.2	0.0	0.7	0
	S42IL-144	33.9	50.1	16.2	2.1	0.0	0.4	1
	S42IL-153	60.7	68.6	7.9	3.5	0.0	0.2	2
	S42IL-110	89.5	97.8	8.3	2.2	0.1	0.5	3
	S42IL-175	118.0	149.5	31.5	4.6	2.3	1.2	5
3H	S42IL-177	23.9	62.7	38.7	10.0	2.1	0.6	7
	S42IL-154	39.6	83.1	43.5	8.5	2.0	1.1	2
	S42IL-111	43.1	55.2	12.1	6.3	0.9	0.3	2
	S42IL-112	59.0	90.9	31.9	2.9	2.7	0.6	1
	S42IL-155	59.0	83.1	24.1	6.1	1.2	0.5	3
	S42IL-179	59.0	86.4	27.4	4.3	0.0	0.3	1
	S42IL-181	69.9	90.9	21.0	1.0	0.8	0.6	0
	S42IL-114	75.9	144.9	69.0	3.0	2.3	0.6	3
	S42IL-140	86.2	148.2	62.0	4.3	0.2	1.2	0
	S42IL-159	88.2	109.2	21.0	2.5	0.0	0.2	1
	S42IL-158	100.3	109.8	9.6	2.0	0.6	0.1	1
	S42IL-148	100.3	131.6	31.3	3.1	0.1	0.5	2
	S42IL-113	120.7	142.2	21.5	1.6	0.0	0.1	0
	S42IL-115	120.7	155.0	34.3	3.3	0.0	0.3	1
	S42IL-160	120.9	137.9	17.0	1.2	0.4	0.8	1
	S42IL-161	139.6	154.9	15.3	1.0	0.0	0.7	0
4H	S42IL-116	1.1	40.0	39.0	3.5	0.0	0.1	4
	S42IL-117	17.8	49.9	32.0	1.7	0.2	0.8	1
	S42IL-118	35.9	54.6	18.7	3.2	0.0	0.4	1
	S42IL-119	35.9	81.2	45.3	6.7	0.0	0.4	1
	S42IL-120	35.9	57.5	21.6	2.5	0.4	0.5	0
	S42IL-164	35.9	69.3	33.4	4.7	0.8	0.6	1
	S42IL-184	40.0	51.0	11.0	0.9	0.6	0.5	0
	S42IL-145	43.3	49.9	6.6	1.9	0.0	0.4	1
	S42IL-162	45.7	81.2	35.6	5.7	0.0	0.7	0
	S42IL-121	51.9	81.2	29.3	5.6	0.7	0.7	2
	S42IL-146	55.7	81.2	25.5	1.6	1.6	0.3	0
	S42IL-166	60.7	81.2	20.5	2.9	0.0	0.3	1
	S42IL-123	85.6	111.3	25.8	4.7	0.0	0.6	2
	S42IL-185	85.6	111.3	25.8	2.4	0.0	0.3	1
	S42IL-124	110.2	115.2	5.0	1.4	0.1	0.3	2
5H	S42IL-125	51.5	81.3	29.9	1.2	0.9	0.5	1
	S42IL-173	51.5	98.9	47.4	2.1	0.0	0.5	0
	S42IL-147	73.3	81.3	8.0	0.5	0.4	0.4	1
	S42IL-126	76.2	120.3	44.2	2.5	0.0	0.6	1
	S42IL-176	81.3	140.1	58.8	7.0	1.0	0.8	3
	S42IL-127	138.5	162.5	24.0	3.8	0.2	0.3	2
6H	S42IL-156	0.3	91.7	91.4	10.2	2.7	0.4	3
	S42IL-149	30.0	51.0	21.0	2.6	0.1	0.2	3
	S42IL-128	38.0	74.6	36.6	9.2	0.4	0.4	2
	S42IL-152	46.2	50.8	4.6	3.1	0.2	1.0	4
	S42IL-186	46.2	63.5	17.2	5.2	2.0	0.5	1
	S42IL-129	47.5	79.6	32.1	8.5	0.5	0.3	0
	S42IL-150	47.5	52.2	4.7	1.9	0.0	0.4	1
	S42IL-130	59.9	105.2	45.3	5.4	2.2	0.4	4
	S42IL-151	59.9	66.8	6.9	0.7	0.5	3.3	2
	S42IL-163	78.1	99.2	21.0	1.9	0.2	0.4	1
	S42IL-131	87.9	108.3	20.4	2.0	0.0	0.6	1
	S42IL-132	94.9	108.3	13.4	1.4	0.0	0.4	1
	S42IL-122	103.8	126.6	22.8	3.7	0.4	0.4	4
7H	S42IL-187	4.1	37.6	33.5	3.5	0.4	0.8	2
	S42IL-133	12.7	37.6	24.9	3.5	0.5	0.4	4
	S42IL-134	37.6	68.4	30.8	2.6	0.2	1.0	1
	S42IL-135	67.8	118.5	50.7	6.1	0.0	0.3	0
	S42IL-167	67.8	74.4	6.7	3.4	0.4	0.4	2
	S42IL-168	67.8	75.1	7.3	3.3	1.2	0.4	2
	S42IL-169	67.8	81.5	13.7	4.1	1.3	0.6	3
	S42IL-170	74.4	79.5	5.1	0.8	1.0	1.0	2
	S42IL-171	76.6	92.1	15.5	1.8	0.5	0.6	3
	S42IL-136	84.6	110.8	26.3	2.0	0.1	0.4	2
	S42IL-137	86.0	127.5	41.6	5.8	0.3	0.2	4
	S42IL-183	99.8	126.7	26.9	2.2	0.0	0.4	1
	S42IL-138	110.8	141.1	30.2	3.3	1.6	0.1	1
	S42IL-139	129.5	141.1	11.5	1.0	0.0	0.6	0

^1^ positions of SNPs are based on Comadran et al. 2012

^2^ Percentage of Hsp, heterozygous, and missing SNPs per genotype

### Drought treatment effect

For the traits YLD, BIO, TGW, GEA, and GWE the drought treatment significantly reduced the trait values compared to the well-watered control treatment (Table 2, S2 Table). As expected, the drought stress treatment limited grain filling indicated by a 28% reduction in the TGW of Scarlett. For all traits, there was a significant genotype main effect but no significant genotype by treatment interaction.

**Table 2:** Descriptive statistics for traits measures in S42IL population per treatment.

	Drought				Well-watered				
	N	Mean	Range	CV^a^	N	Mean	Range	CV	P-value^b^
BIO [g]	584	10.31	6.3–12.0	36.7	574	10.65	6.9–12.1	38.5	***
GEA	583	22.21	14.9–25.5	13.9	576	22.80	15.2–27.3	16.7	***
GWE [g]	583	0.64	0.5–0.8	28.3	576	0.81	0.6–1.0	31.1	***
HEA [days]	1176	54.88	34.3–59.8	11.9	na	na	na	na	na
HEI [cm]	1159	90.13	79.7–97.2	8.7	na	na	na	na	na
TGW [g]	583	28.57	23.2–35.3	24.3	576	35.43	30.2–42.8	24.1	***
YLD [g]	583	5.49	4.4–6.9	32.1	576	7.41	5.7–9.5	37.6	***

^a^ Coefficient of variation in %

^b^ Indicates if treatment effect was significant with p < 0.001 ‘***’

### Trait correlations

Overall similar correlations were detected between the seven measured traits under well-watered and drought treatments (S3 Table). The yield components TGW, GEA and GWE all showed a significant positive correlation with YLD. HEA was not correlated with YLD, however it was negatively correlated with TGW and positively correlated with GEA.

### S42IL by trait associations and QTL detection

For the seven traits measured a total of 100 significant line by trait associations were detected in the set of 49 S42ILs. From the five traits measured following the well-watered and drought stress treatment, 46 and 25 line by trait associations were detected within each treatment group respectively, with 15 associations significant in both treatments. From the two traits, HEI and HEA that were measured before the treatment began, 29 line by trait associations were identified.

**Fig 2:**
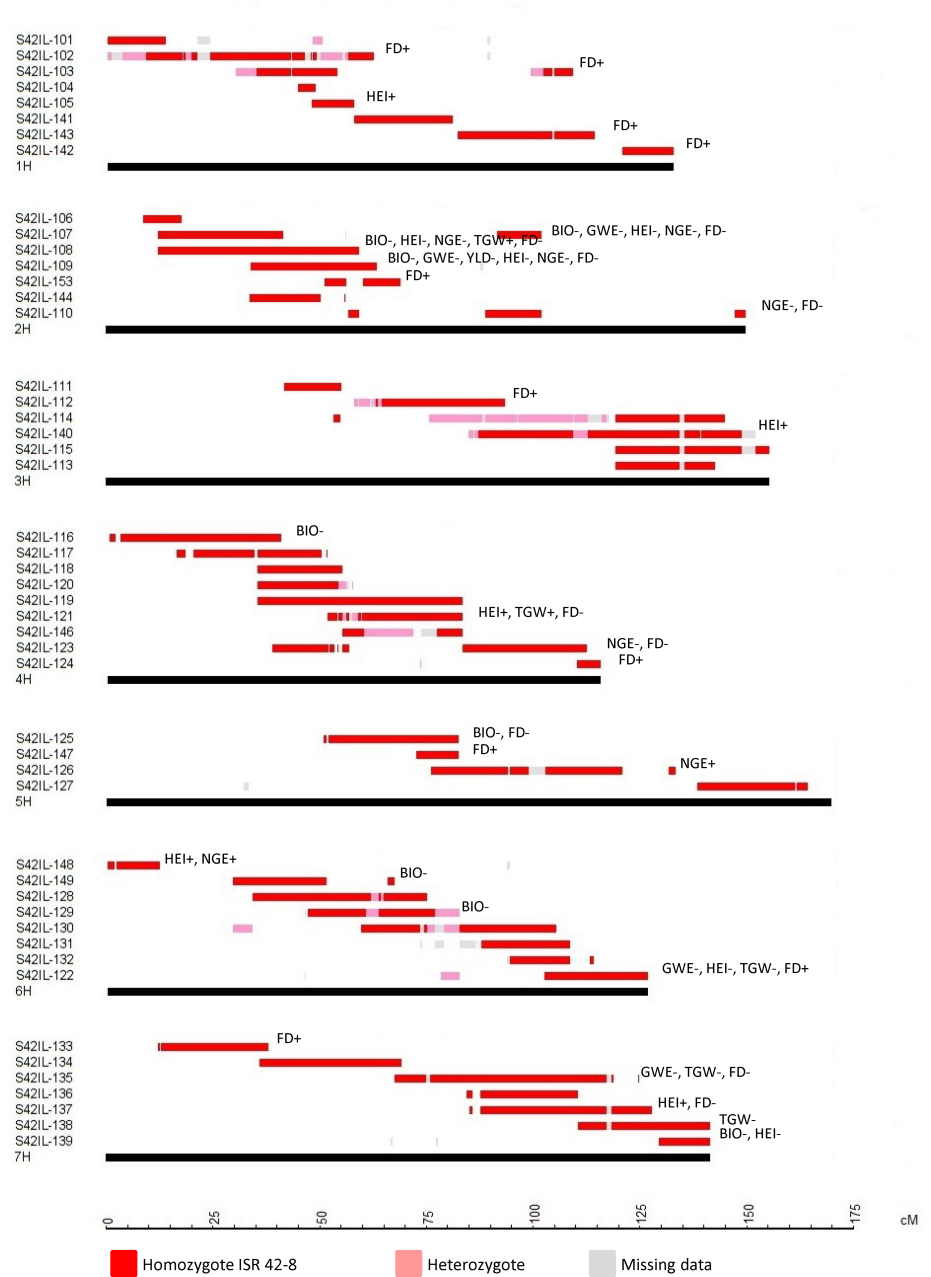
Genetic map with selected S42IL introgressions, QTL are placed right to the S42ILs, indicated by trait abbreviations (see Table 1). The sign indicates an increasing (+) or decreasing (-) *Hsp* effect.

Taking into account that several of the S42ILs contain overlapping or flanking *Hsp* introgression, a total of 22 QTL were identified that were either detected as a line main effect (e.g. across both treatments) or as a line by drought treatment effect, only present under drought treatment (Fig 2 and Table 3). In addition, 19 QTL were identified for the traits HEI and HEA. The QTL are summarised below.

**Table 3:** List of S42ILs significantly different from Scarlett under drought stress for each trait.

Trait	QTL	S42_IL	Chr.^a^	Position^a^	Effect^b^	Mean	Diff ^c^	RP %^d^	Candidate genes^e^
BIO		Scarlett				10.9			
	QBIO.2H.1	S42IL-107	2H	12.5–41.2	a	6.3	-4.7	-42.8	*Ppd-H1*
		S42IL-108	2H	12.5–59.1	d	8.5	-2.5	-22.8	*Ppd-H1*, *HvFt4*
	QBIO.2H.2	S42IL-109	2H	33.9–62.7	d	8.9	-2.1	-18.7	*HvFt4*
	QBIO.4H.3	S42IL-116	4H	1.1–40.0	d	9.4	-1.5	-14.1	
	QBIO.5H.4	S42IL-125	5H	51.5–81.3	d	9.4	-1.6	-14.4	
	QBIO.6H.5	S42IL-149	6H	30.0–51.0	d	9.4	-1.5	-13.9	
		S42IL-129	6H	47.5–79.6	a	8.8	-2.2	-19.6	*HvCO2*
	QBIO.7H.6	S42IL-139	7H	129.5–141.1	a	9.3	-1.6	-15.0	
GWE		Scarlett				0.7			
	QGWE.2H.1	S42IL-107	2H	12.5–41.2	a	0.5	-0.2	-29.0	*Ppd-H1*
	QGWE.2H.2	S42IL-109	2H	33.9–62.7	a	0.5	-0.2	-28.7	*HvFt4*
	QGWE.6H.3	S42IL-122	6H	103.8–126.6	a	0.5	-0.2	-22.3	
	QGWE.7H.4	S42IL-135	7H	67.8–118.5	d	0.5	-0.1	-21.7	*HvCO1*
YLD		Scarlett				5.9			
	QYLD.2H.1	S42IL-109	2H	33.9–62.7	a	4.4	-1.6	-26.3	
HEI		Scarlett				90.5			
	QHEI.1H.1	S42IL-105	1H	48.1–57.3	a	96.5	5.9	6.5	
	QHEI.2H.2	S42IL-107	2H	12.5–41.2	a	79.7	-10.8	-12.0	*Ppd-H1*
	QHEI.2H.2 and 3	S42IL-108	2H	12.5–59.1	a	83.3	-7.3	-8.1	*Ppd-H1*, *HvFt4*
	QHEI.2H.3	S42IL-109	2H	33.9–62.7	a	85.3	-5.2	-5.8	*HvFt4*
	QHEI.3H.4	S42IL-140	3H	86.2–148.2	a	96.4	5.9	6.5	
		S42IL-121	4H	51.9–81.2	a	96.1	5.5	6.1	
		S42IL-148	6H	0.3–11.3	a	95.3	4.7	5.2	
	QHEI.6H.5	S42IL-122	6H	103.8–126.6	a	86.7	-3.9	-4.3	
	QHEI.7H.6	S42IL-137	7H	86.0–127.5	a	97.2	6.6	7.3	
	QHEI.7H.7	S42IL-139	7H	129.5–141.1	a	86.6	-4.0	-4.4	
GEA		Scarlett				22.3			
	QGEA.2H.1	S42IL-107	2H	12.5–41.2	a	14.9	-7.4	-33.3	*Ppd-H1*
		S42IL-108	2H	12.5–59.1	d	20.0	-2.3	-10.3	*Ppd-H1*, *HvFt4*
	QGEA.2H.2	S42IL-109	2H	33.9–62.7	a	16.8	-5.5	-24.5	*HvFt4*
		S42IL-110	2H	89.5–97.8	a	17.9	-4.4	-19.7	
	QGEA.4H.3	S42IL-123	4H	85.6–111.3	a	19.6	-2.7	-12.0	
	QGEA.5H.4	S42IL-126	5H	76.2–120.3	d	24.6	2.3	10.2	
	QGEA.6H.5	S42IL-148	6H	0.3–11.3	d	25.5	3.2	14.5	
TGW		Scarlett				29.3			
	QTGW.2H.1	S42IL-108	2H	12.5–59.1	d	35.3	6.0	20.4	*Ppd-H1*, *HvFt4*
	QTGW.4H.2	S42IL-121	4H	51.9–81.2	d	34.3	5.0	16.9	
	QTGW.6H.3	S42IL-122	6H	103.8–126.6	a	24.1	-5.3	-17.9	
	QTGW.7H.4	S42IL-135	7H	67.8–118.5	d	23.2	-6.1	-20.9	*HvCO1*
	QTGW.7H.5	S42IL-138	7H	110.8–141.1	d	24.9	-4.4	-15.0	*HvFT3*
HEA		Scarlett			a	55.6			
	QHEA.1H.1	S42IL-102	1H	0.2–62.3	a	58.3	2.8	5.0	
		S42IL-103	1H	32.2–53.7	a	58.9	3.3	6.0	
	QHEA.1H.2	S42IL-143	1H	82.6–112.3	a	57.3	1.7	3.0	
		S42IL-142	1H	122.1–132.7	a	57.9	2.3	4.2	
	QHEA.2H.3	S42IL-107	2H	12.5–41.2	a	34.5	-21.1	-37.9	*Ppd-H1*
		S42IL-108	2H	12.5–59.1	a	34.3	-21.3	-38.2	*Ppd-H1*, *HvFt4*
	QHEA.2H.4	S42IL-109	2H	33.9–62.7	a	52.6	-3.0	-5.4	*HvFt4*
		S42IL-110	2H	89.5–97.8	a	49.5	-6.1	-10.9	
		S42IL-153	2H	60.7–68.6	a	57.4	1.8	3.3	
	QHEA.3H.5	S42IL-112	3H	59.0–90.9	a	57.2	1.6	2.9	
	QHEA.4H.6	S42IL-121	4H	51.9–81.2	a	53.6	-2.0	-3.6	
		S42IL-123	4H	85.6–111.3	a	53.5	-2.1	-3.8	
	QHEA.4H.7	S42IL-124	4H	110.2–115.2	a	58.2	2.6	4.7	*VRN-H2*
	QHEA.5H.8	S42IL-125	5H	51.5–81.3	a	53.8	-1.8	-3.2	
	QHEA.5H.9	S42IL-147	5H	73.3–81.3	a	57.3	1.8	3.2	
	QHEA.6H.10	S42IL-122	6H	103.8–126.6	a	57.2	1.6	2.9	
	QHEA.7H.11	S42IL-135	7H	67.8–118.5	a	53.8	-1.8	-3.2	*HvCO1*
		S42IL-137	7H	86.0–127.5	a	54.1	-1.5	-2.7	
	QHEA.7H.12	S42IL-133	7H	12.7–37.6	a	59.8	4.3	7.7	*VRN-H3*

^a^ Positions according to Comadran et al. 2012

^b^ a: across treatments; d: under drought treatment

^c^ The absolute difference in the trait values under drought stress between the S42IL and Scarlett.

^d^ Relative performance: RP[IL] = (LSMEANS[IL]–LSMEANS [Scarlett]) x 100/LSMEANS[Scarlett]

^e^ Candidate genes with effects on flowering time and agronomic traits mapped by Wang et al. 2010

#### Yield

Only S42IL-109 was significantly different to Scarlett under drought stress. This effect was attributed to a single QTL on chromosome 2H position 63–110 cM that reduced YLD by 26% compared to Scarlett.

#### Biomass

In total eight S42ILs showed significant difference in BIO compared to Scarlett under drought stress, which could be attributed to six QTL. The largest effect was associated with the QTL QBIO.2H.1 on chromosome 2H, reducing biomass by up to 42% in S42IL-107.

#### Thousand grain weight

Five line by trait associations were identified for TGW under drought stress. All *Hsp* introgressions in these lines were independent, therefore five unique QTL were identified. Lines S42IL-108 and -121 showed a 20% and 16% increase in TGW under drought stress respectively, whereas the TGW of lines S42IL-135 and -138 was reduced by 20% and 15% under drought stress respectively compared to Scarlett. Notably, the TGW of these three lines was not significantly different to Scarlett under the well-watered treatment.

#### Number of grains per ear

Seven line by trait associations were detected from which five QTL were identified for GEA. The largest effect was due the QTL QGEA.2H.1 on chromosome 2H that reduced GEA by up to 33% in line S42IL-107 compared to Scarlett. QTL detected on 5H and 6H increased GEA by 10 and 14% respectively compared to Scarlett.

#### Grain weight per ear

Four S42IL by trait associations were detected, which could be attributed to four QTL on chromosomes 2H, 4H, and 6H. All QTL reduced GWE ranging from 21–29% compared to Scarlett.

### Days to heading

In total 19 significant S42IL by trait associations were detected. These effects were contributed by 12 QTL located on all seven barley chromosomes. The largest effect was due to the 2H QTL QHEA.2H.3 that reduced HEA by 21 days compared to Scarlett, whereas the QTL QHEA.1H.1 extended HEA by up to three days.

#### Plant height

Ten line by trait associations were detected for HEI. Due to overlapping introgressions the ten associations were summarized to seven QTL. All QTL were detected as line main effects on all chromosomes but 5H. In five S42ILs, the *Hsp* alleles led to increased plant height of 5.2 to 7.3%. Plant height reduction for the other five genotypes was between 4.3 and 12%.

## Discussion

### Genotyping and S42IL map

The S42IL library was genotyped with the Infinium 9k iSelect chip, which comprises of 7,864 SNPs. Of these SNPs 2,487 (31.6%) were polymorphic in the S42IL library and mapped by Comadran et al. [34]. A more recent consensus map was published by Muñoz-Amatriaín et al. [37]. We decided to use the older map because it contained more mapped SNP markers polymorphic in the S42IL library. When 73 S42ILs were previously genotyped with the Infinium BOPA1 SNP chip, the results were similar. Of 1,536 SNPs 636 (41.4%) were polymorphic between the two parents [22]. The amount of polymorphism also corresponds to findings in the wild barley NAM population HEB-25, made of parallel crosses between one spring barley cultivar (Barke) and 25 wild barley donor accessions [31]. The authors reported of 5,709 SNPs (72.6%), which were informative in at least one sub-family of HEB-25, using the same barley 9k iSelect chip. On average, 2,642.8 SNPs (33.6%) were polymorphic per HEB-25 sub-family (Maurer, pers. comm.).

The share of polymorphism seems relatively low compared for instance to 3,973polymorphic SNPs in the Morex and Barke RIL population used to construct an iSelect map. One reason for the lower number of polymorphisms in the S42IL library might be that the assay was developed based on ten different cultivars without any wild barley accessions [34]. Ascertainment bias in favor of *Hv* [38], therefore, might be one reason that relatively fewer SNPs were detected between Scarlett and ISR42-8.

The set of 2,487 SNPs was further manually curated. Fifty SNPs were removed since they showed double crossover events, which were unlikely to be correct. Only about 70% of the SNP markers were mapped. Therefore, of the selected 3,009 SNPs, only 2,487 could be used to construct the map of the S42IL library. On average, each S42IL carries 1.7 additional introgressions. Out of the 83 S42ILs 68 (82%) showed an additional introgression. The numbers are increased compared to the BOPA1 data with 1.5 introgressions on average and 72.6% of the S42ILs carrying at least one additional introgression [22]. With denser genotypic information, more additional introgressions were detected. This was already observed by Honsdorf et al. [27]. While Schmalenbach et al. [22] reported that 20 out of 73 S42ILs (27.4%) carry a single introgression, Honsdorf et al. [27] reported that only 25.9% corresponding to 14 out of 54 S42ILs carry a single introgression. In the new iSelect based map, 15 out of 83 S42ILs carry only one introgression (18.1%). The results show that the higher the number of SNP markers and genotypes is, the higher is also the number of additionally detected introgressions.

### Drought experiments

For the five traits measured after the onset of drought treatment, the ANOVA showed a clear effect of treatment on trait performance. Under drought treatment, trait performance was reduced. No statistically significant interaction effect between genotype and treatment was observed. This is in accordance with the plant breeding axiom, that the best genotypes perform best under most environments [39]. When a set of S42ILs was tested under moderate drought stress at juvenile growth stage a similar observation was made [28]. When severe drought stress was applied, however, the axiom did not hold true anymore. Under severe drought stress the S42ILs showed interaction with the treatment and the best genotypes under moderate stress and well-watered conditions did not perform best under severe stress [27]. Blum [39] points out that at a certain stress level, cross over effects arise and genotypes with lower yield under non-stress conditions outperform high yielding varieties.

### QTL in the S42IL library

In total 40 QTL for seven investigated traits were detected in this study. Half of these QTL had already been detected in previous studies on the S42IL library. Most QTL (12) were detected for flowering time. Only one QTL was detected for yield. In the majority of the cases (26 out of 40) the *Hsp* allele had a trait reducing effect. While a reduced trait expression is not necessarily negative, in this study the *Hsp* allele was the unfavorable allele in most cases. Since wild barley is not adapted to agricultural production it is not surprising, that for most detected effects the *Hsp* allele has a negative effect. Twenty-eight of the 49 investigated S42ILs revealed differences in trait performance compared to Scarlett. S42IL-107, -108, and -109 contain overlapping introgressions and showed reduced trait performance for the traits BIO, HEI, GEA, and HEA. S42IL-107 and -108 contain the flowering gene *Ppd-H1* and S42IL-108 and -109 contain flowering time gene *FT4* [40]. These differences observed between S42ILs and Scarlett may be attributed to contrasting growth habits of the parents. While Scarlett is a spring barley cultivar, insensitive to photo period, ISR42-8 is a winter type wild barley, exhibiting a strong response to a photo period extension. The wild barley allele of *Ppd-H1* causes early flowering. The shortened period in which the generative growth phase is reached may lead to reduced biomass production in S42IL-107 and -108. Both lines required 21 days less to flower than Scarlett. However, biomass was reduced to different extents in both genotypes. In S42IL-107, biomass was reduced by almost 43%, in S42IL-108 it was reduced by 23%. Grains per ear were likewise reduced stronger in S42IL-107 (-33%) than in S42IL-108 (-10%). S42IL-108 and -109 both harbor the same *Hsp* allele for *FT4*. Differences in biomass reduction were more similar between the two genotypes (23 and 19%, respectively) as compared to S42IL-107. While the shortened growth period might have an influence on biomass production, it does not entirely explain the low biomass production of S42IL-107 since S42IL-108 had similar flowering time but almost 50% less biomass reduction compared to S42IL-107. Interestingly, the genotype shows increased biomass production under severe drought stress at the juvenile development stage [27]. The rapid development seems beneficial under circumstance where water becomes scarce early during the growth period. When water shortage arises later during the growth period, however, early flowering and fast development is less advantageous.

S42IL-121 is an example for a genotype where the *Hsp* allele had positive effects on trait performance. The genotype flowered slightly earlier than Scarlett, was slightly taller and its TGW was increased under drought treatment by 17%. In previous experiments on drought tolerance during juvenile development S42IL-121 showed improved biomass production across drought and control treatment [28]. S42IL-121 is a promising candidate for the improvement of barley under drought stress at juvenile growth stage and during grain filling.

The S42IL library was investigated in several experiments. In the following QTL detected in this study are compared to QTL described in previous studies on the S42IL library. In addition, references to similar studies are given.

#### Biomass

Complete biomass has been investigated in two previous S42IL studies on juvenile drought tolerance [27,28]. S42IL-129 showed reduced biomass production in all three studies under control and moderate drought treatment. However, under severe drought stress S42IL-129 does not reduce biomass production compared to Scarlett [27]. It thus seems that the introgression that appears to confer a negative effect under the applied drought treatment has positive effects under more severe stress conditions. Lakew et al. [41] conducted field experiments in which they studied drought stress tolerance of wild barely introgression lines. They detected QTL for biomass yield on chromosomes 2H and 6H. Arriagada et al. [42] and Mora et al. [43] tested 137 barley recombinant chromosome substitution lines, derived from a cross between wild barley accession Caeserea 26–24 and malting barley Harrington, in Mediterranean environments in Chile under rain fed conditions. They detected QTL for biomass on 2H, 5H, and 7H. These QTL might correspond to QTL QBIO.2H.1, QBIO.2H.2, QBIO.5H.4, QBIO.6H.5, and QBIO.7H.6 detected in the present study.

#### Grain weight per ear

Four QTL for GWE were detected on chromosomes 2H, 6H, and 7H. In all cases, the *Hsp* introgressions reduced trait performance. Few studies investigated grain weight per ear. Ren et al. [44] detected two QTL for this trait on chromosomes 4H and 7H. There might be an overlap for the QTL on 7H between the two studies. Mikolajczak et al. [45] tested barley recombinant inbred lines under water limited conditions in greenhouse experiments and detected QTL for grain weight per ear on 2H which might correspond to QTL QGWE.2H.2 detected in our study.

#### Yield

Yield was reduced by 26% in S42IL-109. This QTL was not detected in any previous study on the S42IL library. However, the genotype had reduced trait performance for yield components as well. Grain weight per ear and GEA were reduced compared to Scarlett. Moreover, the genotype produced less biomass and was shorter. The reduced overall performance may explain the reduced yield. Abou-Elwafa [46] conducted an association mapping study with 107 wild barley genotypes. The experiment was carried out as pot experiment and single plant yield was measured. Mikolajczak et al. [45] measured grain weight per plant as well. Both studies found QTL for single plant yield on chromosome 2H. These QTL may correspond to the QTL detected in our study.

#### Plant height

Effects on plant height were small, ranging from 4% to a maximum of 12% in S42IL-107. In this genotype, height was reduced by 12%, which can be explained by the early flowering *Hsp* allele of *Ppd-H1* present in the genotype. Six out of the seven QTL were already detected in previous studies on the S42IL library. The QTL in S42IL-107, for instance, was described by Schmalenbach et al. [23], Wang et al. [40], and Honsdorf et al. [27]. In the latter publication, juvenile plants were studied. The results show that this QTL is very stable and even visible in experiments after a short time, in this case five weeks. This is also true for the five remaining HEI QTL. Even though effects were small, they are very stable across different growth conditions ranging from hydroponic cultures [25] to greenhouse cultivation and field trials. In our study QTL for height were detected on six out of seven chromosomes. Similarly Abou-Ewafa [46] detected QTL for plant height on all seven chromosomes in a set of 107 wild barley accessions. This might confirm findings in our study and shows the complexity of plant height control.

#### Number of grains per ear

Three QTL in which the *Hsp* allele caused a decrease in GEA had been reported in earlier studies [23,26,40]. In two cases, the *Hsp* alleles increased GEA by 10 and 15%, respectively. The favorable effects detected on chromosomes 5H and 6H in S42IL-126 and -148, respectively, had not been described before. Lakew et al. [41] detected a positive *Hsp* effect on chromosome 5H. This might be the same QTL underlying the effect in S42IL-126. Mikolajczak et al. [45] detected QTL for number of grains per ear on 2H, 3H, 5H, and 7H. The QTL on 2H and 5H might confirm findings of our study.

#### Thousand grain weight

At two QTL on chromosomes 2H and 4H wild barley alleles increased trait performance by 20 and 17%, respectively. These results verify observations by Schmalenbach et al. [23] and Wang et al. [40]. Reduced trait performance caused by the *Hsp* allele on chromosome 6H in S42IL-122 was as well reported in [23]. Two new QTL, detected on chromosome 7H, caused reduction of TGW by 21 and 15%. Lakew et al. [41] reported four QTL for TGW on chromosome 7H. Two of them might be located in the region where QTL were detected in the present study. However, for QTL described in the mentioned study the *Hsp* allele caused increased TGW. Von Korff et al. [16] and Saal et al. [47] tested the S42 population, the population of which the S42IL library was derived of. They detected trait-reducing effects in the same chromosomal region on 7H where the effects in this study were mapped. QTL effects on 2H, 4H, and 7H were also detected by Arriagada et al. [42] and Mora at al. [43].

#### Days to heading

In this study twelve QTL for flowering time were detected. Since drought treatment was applied after flowering no treatment effects were observed. In seven out of twelve QTL, the *Hsp* allele delayed flowering time. In general, QTL effects were small, causing plants to flower two or three days earlier or later than Scarlett. Three QTL with greater effects were observed. In HEA.7H.12, detected in genotype S42IL-133, the *Hsp* allele caused a flowering time delayed by four days. This QTL was already described in [23] and [40]. In S42IL-110, the *Hsp* allele at HEA.2H.4 caused six days earlier flowering. The same QTL, however, had smaller effects in two other genotypes. The effect had already been detected in a previous study [23]. The largest effect was detected in S42IL-107 and -108. Flowering time in the two genotypes was 21 days ahead of Scarlett. They carry an *Hsp* introgression on chromosome 2H including the flowering time gene *Ppd-H1*. The dominant allele is responsible for early flowering date. This was observed in several studies [23,26,40]. *Ppd-H1* was mapped in S42IL-107 and -108 [40].

## Conclusion

In this study, wild barley introgression lines were tested for drought stress response during grain filling in four greenhouse experiments. Several QTL where the exotic *Hsp* allele had a positive effect on trait performance were detected. This confirms the potential value of the wild progenitor to improve barley performance. In particular, two QTL for GEA on 5H and 6H and two QTL for TGW on 2H and 4H are promising candidates to improve yield under drought stress. Especially, S42IL-121 is a promising candidate. The genotype had improved TGW under drought. In a previous greenhouse study on drought during juvenile development stage the line had improved vegetative growth and biomass. Moreover, improved biomass production was also observed for this genotype under non-stress field conditions. S42IL-121, thus, might be an interesting candidate for improving biomass production and TGW of barley cultivars.

It might also be of interest to clone the wild barley genes responsible for the most promising QTL identified in this study, given the current advent of new genomic tools available for barley genetics. A map-based cloning of the TGW QTL might be feasible making use of the new barley RefSeq 1.0 genome sequence of the International Barley Sequencing Consortium [48] and the recent advances in CRISPR/Cas9 genome editing [49,50].

## Supporting information

S1 Table SNP map with 3,059 markers for 83 S42ILs based on the Illumina iSelect chip. (XLSX)

S2 Table Phenotype raw data. (XLSX)

S3 Table Pearson’s correlation coefficient (r) between seven traits measured in the S42IL population under the well-watered and drought treatments. (XLSX)
